# *Carica papaya* extract in dengue: a systematic review and meta-analysis

**DOI:** 10.1186/s12906-019-2678-2

**Published:** 2019-10-11

**Authors:** Senaka Rajapakse, Nipun Lakshitha de Silva, Praveen Weeratunga, Chaturaka Rodrigo, Chathurani Sigera, Sumadhya Deepika Fernando

**Affiliations:** 1 Department of Clinical Medicine, Faculty of Medicine, University of Colombo, 25, Kynsey Road, Colombo 8, Colombo, Sri Lanka; 2 Department of Clinical Medicine, Faculty of Medicine, General Sir John Kotelawela Defence University, Rathmalana, Sri Lanka; 3 Department of Pathology, School of Medical Sciences, University of New South Wales, Sydney, NSW Australia; 4 Department of Parasitology, Faculty of Medicine, University of Colombo, Colombo, Sri Lanka

**Keywords:** Dengue, Thrombocytopaenia, *Carica papaya*, Papaya leaf extract

## Abstract

**Background:**

*Carica papaya* (CP) extract is becoming popular as an unlicensed herbal remedy purported to hasten recovery in dengue infection, mostly based on observations that it may increase platelet counts. This systematic review and meta-analysis aims to critically analyze the evidence from controlled clinical trials on the efficacy and safety of CP extract in the treatment of dengue infection.

**Methods:**

PubMed, LILACS and Google Scholar were searched for randomized or non-randomized trials enrolling patients with suspected or confirmed dengue where CP extract was compared, as a treatment measure, against standard treatment. Recovery of platelet counts as well as other clinical indicators of favourable outcome (duration of hospital stay, prevention of plasma leakage, life threatening complications, and mortality) were assessed.

**Results:**

Nine studies (India-6, Pakistan-1, Indonesia-1, Malaysia-1) met the inclusion criteria. Seven studies showed an increase in platelet counts in patients receiving CP extract, while one study showed no significant difference between the two groups, and direct comparison was not possible in the remaining study. Serious adverse events were not reported. CP extract may reduce the duration of hospital stay (mean difference − 1.98 days, 95% confidence interval − 1.83 to − 2.12, 3 studies, 580 participants, low quality evidence), and cause improvement in mean platelet counts between the first and fifth day of treatment (mean difference 35.45, 95% confidence interval 23.74 to 47.15, 3 studies, 129 participants, low quality evidence). No evidence was available regarding other clinical outcomes.

**Conclusions:**

The clinical value of improvement in platelet count or early discharge is unclear in the absence of more robust indicators of favourable clinical outcome. Current evidence is insufficient to comment on the role of CP extract in dengue. There is a need for further well designed clinical trials examining the effect of CP on platelet counts, plasma leakage, other serious manifestations of dengue, and mortality, with clearly defined outcome measures.

## Introduction

Dengue is an arboviral infection transmitted by mosquitoes of the *Aedes* species. It is a disease with global implications, resulting in considerable morbidity and mortality. Transmission occurs in at least 128 countries, and almost 4 billion people are at risk [1]. Data from the Global Burden of Disease Study 2013 showed that the incidence of dengue has markedly increased over the years, from 8.3 million cases in 1990 to a staggering 58.4 million cases in 2013 [2]. The annual average number of deaths due to dengue from 1990 to 2013 was estimated at 9221. It is currently estimated that around 390 million dengue infections occur annually, and that 96 million of these result in clinical disease [3]. South and South East Asia account for a major proportion of the global burden of dengue, with an estimated mortality of 8.49 per million population in 2013 [2].

Dengue fever occurs due to infection by four distinct serotypes (DEN 1–4), and has diverse manifestations, ranging from an uncomplicated febrile illness to serious disease with organ dysfunction. In the more severe forms, plasma leakage gives rise to shock and organ failure; life threatening haemorrhage can also occur. Unusual organ manifestations of dengue are also increasingly reported, and comprise the expanded dengue syndrome [4]. Mortality in dengue is most often due to shock, intractable multi-organ dysfunction, or uncontrollable bleeding. Excessive fluid therapy is also known to contribute to mortality in patients with plasma leakage, due to the development of pulmonary oedema during the recovery phase.

Despite many years of extensive research, specific therapeutic modalities for dengue remain in their infancy. Immunosuppression, in the form of corticosteroids [5] or immunoglobulins, [6] has not shown benefit. Several antiviral agents and other host immune modulators are in the early stages of clinical evaluation, and will not be available for clinical use in the foreseeable future [7]. Several dengue vaccines are in development, and some already licensed for use, but these do not show convincing evidence of benefit across all age groups, and do not confer immunity for all serotypes [8]. The mainstay of clinical treatment of dengue is careful fluid management, with close monitoring and supportive care.

The lack of effective therapeutic interventions for dengue has created interest in alternative therapies, i.e., natural and herbal remedies for the disease. *Carica papaya* (CP) leaf extract has recently gained interest in the treatment of dengue, particularly in social media networks, and has shown increasing off-label use in the disease. While the simple extract of papaya leaves is commonly used, there are some commercial preparations containing papaya leaf extract available in certain countries.

CP is a ubiquitous flowering plant in the tropics, with an edible fruit. Originally native to Mexico and South America, it has flourished after introduction to South and South-East Asian countries. The leaves of the plant contain several biologically active compounds, such as papain, caricain, chymopapain, and glycine endopeptidase. These compounds have been shown to improve acidic pH, and cause degradation of pepsin [9]. CP also contains lipase, which is bound to the water-insoluble component of papain [10]. CP leaf extract has been purported to have anti-viral and haematological effects which might have pathophysiological implications for its use as treatment for dengue, such as anti-oxidant and free radical scavenging properties, [11] and improved red cell membrane stabilization [12]. The flavonoids of CP leaf extract have been shown to inhibit a protease involved in viral assembly [13].

During recent epidemics, patients have resorted to the off-label use of CP extract in dengue infection. However intense debate continues regarding the therapeutic efficacy and safety profile of this treatment. This systematic review and meta-analysis aims to critically analyze the available clinical evidence from prospective controlled clinical trials on the efficacy and safety of CP extract in the treatment of dengue infection.

## Methods

### Eligibility criteria

We included prospective clinical trials (randomized or non-randomized) with a control group (placebo or no treatment).

### Information sources and search strategy

We searched PubMed, LILACS and Google Scholar for relevant articles. PubMed and LILACS were searched with the keywords ‘dengue’ in any field and ‘papaya’ in any field without any language, time, or other restrictions. A Google Scholar search was performed with the keywords ‘dengue’ and ‘papaya’ in the title of the article with no other restrictions. References provided in full papers were also used to identify additional papers for review. The last date of the search was 2nd August 2019. We used the software Endnote X7 (Thomson Reuters, Carlsbad, CA 92011, USA) to filter articles. Review Manager (RevMan) version 5 (Cochrane Collaboration) was used to analyze the data.

### Study selection and data collection

SR, PW, NLdS and CR independently screened the abstracts and selected articles reporting controlled studies. Eligible studies were finalized by consensus among all authors.

### Outcomes and data items extracted

Data from individual studies were extracted using a customized data extraction proforma designed by the authors. The data items extracted from each eligible study included: location of the trial, participant demographics, patient characteristics of test and control groups, severity of disease, details of interventions, and outcome measures.

### Selected outcomes

We chose the following outcome measures: mortality, incidence of plasma leakage, shock, minor and major bleeding manifestations, organ complications, changes in platelets counts, haematocrit as surrogate measure of plasma leakage, days of hospitalization, transfusion requirements, need for intensive care, and safety outcomes.

### Risk of bias

We assessed the risks of bias of included studies qualitatively using the Cochrane risk of bias assessment tool [14].

### Summary measures and synthesis of results

When comparative trials were available to combine into a meta-analysis, we analyzed the data using RevMan 5. Dichotomous data were compared with relative risk (RR) and 95% confidence intervals (CI), and continuous data with mean differences. A fixed effect model was used for analysis. We assessed heterogeneity by observing overlapping confidence intervals in the forest plots, chi-square test, and using the I^2^ statistic. An I^2^ statistic greater than 70% was considered as a high level of heterogeneity, and a random effect model was used for the analysis.

## Results

**Fig. 1:**
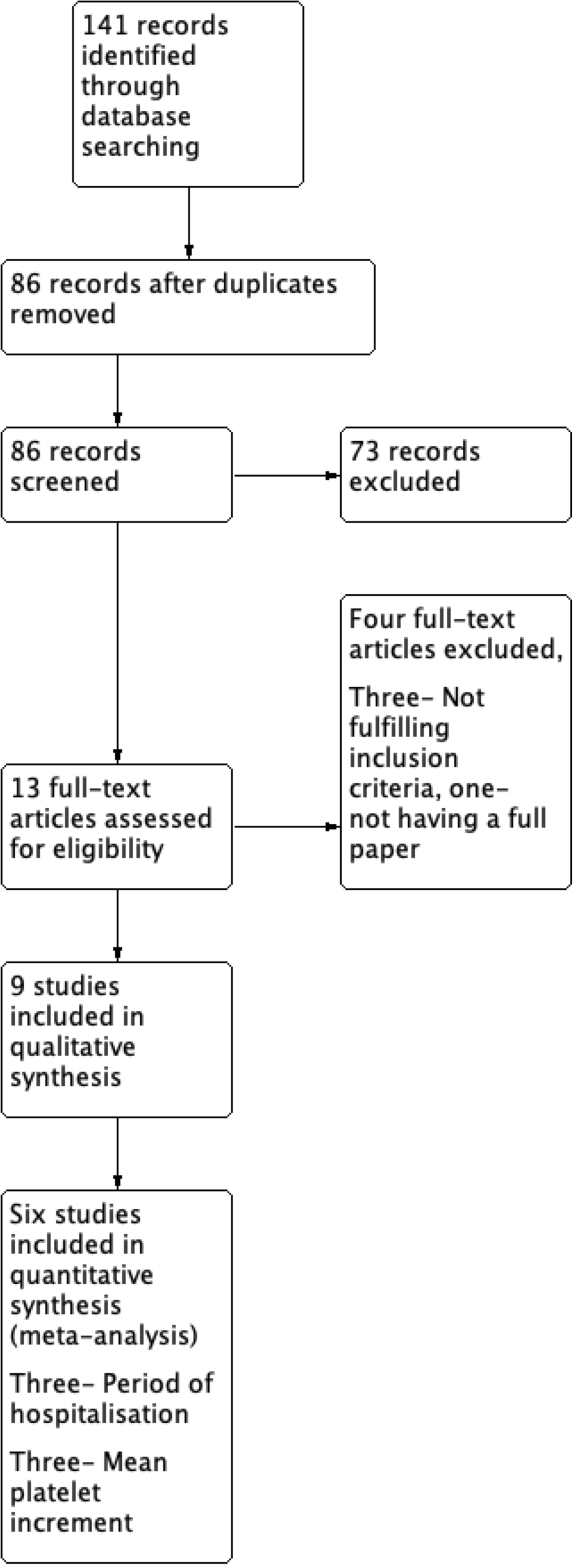
PRISMA flow diagram

**Fig. 2:**
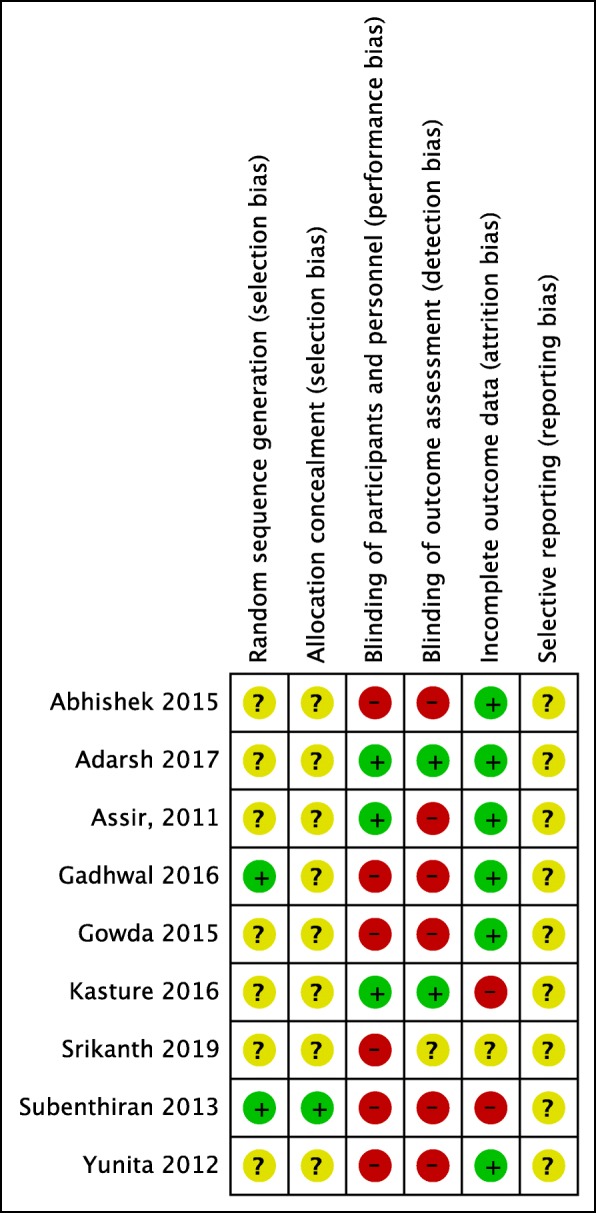
Risk of bias summary

The initial search yielded 36 results from PubMed, 72 results from Google Scholar and 33 from LILACS. After removing duplicates, 86 abstracts were left for review. The PRISMA flow diagram is shown in Fig. 1, and depicts the process of selecting studies for the systematic review and meta-analysis. After screening of abstracts, 13 potential papers were identified. After excluding three studies which did not fulfill inclusion criteria and one study where the full paper could not be obtained despite repeated attempts to contact the authors, 9 papers remained. Nine full text articles were selected for qualitative analysis and only six were selected for quantitative analysis. Fig. 2 summarizes the review authors’ judgments about each risk of bias item for included studies. Assessment of each study with regards to selection bias, performance bias, detection bias, attrition bias and reporting bias is summarized. Risk of bias is graded as low risk, high risk and unclear risk. A summary of all studies is provided in Table 1. In brief, all studies but three were conducted in India (Pakistan-1, Indonesia-1, Malaysia- 1). All studies were mentioned as randomized but seven did not specify the method of random allocation. All the studies recruited young adults or adults (age range 15–60 years) except one which included only paediatric patients (age range 1–12 years). Two studies were double blind and one was single blind.

**Table 1:** Summary of included studies

Study	Methods	Participants	Interventions	Outcomes	Adverse events
Assir et al. [15], 2011	Randomized single blind placebo controlled study	Patients > 14 years with DF or DHF with platelet < 50 × 10^9^(19 intervention, 20 control)	CP leaf extract syrup 5 ml twice a day for four days	Mean platelet count increments after 2–4 days were not statistically significant between the two groups. Mean platelet increment between day 5(4 days after intervention) and baseline was 106 ± 69.16 in the intervention group and 82.3 ± 37.28 in the control group, and not statistically significant.	No significant adverse effects with CP
Yunita et al. [16], 2012	Randomized open-label controlled study	Patients 15–55 years with suspected DF (40 in each arm)	CP leaf extract capsule 1100 mg three times a day	Platelet counts rise more rapid in the intervention group (*p* < 0.05). Haematocrit changes not significant. Period of hospitalization shortened with treatment. Intervention group 3.48 ± 0.6 days, Control group 5.38 ± 0.67 days (*p* < 0.05).	Not reported
Subenthiran et al. [17], 2013	Randomized open label controlled study	Patients 18–60 years with DF or DHF with platelet < 100 × 10^9^(145 in each arm)	Fresh juice from 50 g of CP leaves daily for three days	Mean difference in platelet count at 8 h and after 40 h: intervention group − 16.764 (− 24.566, − 8.964), *p* < 0.001, control group − 7.703 (− 14.055, 1.351) (*p* = 0.018).	Not reported
Abhishek et al. [18], 2015	Randomized open label controlled study	Patients 18–60 years with DF or DHF I, II with platelet count 30 – 100 × 10^9^ (30 in each arm)	CP leaf extract tablets 1100 mg three times a day for five days	Mean platelet count after five days: intervention group 110.71 + 30.57, control group 75.63 + 22.49. Mean difference in platelet count from day 1 to day 5: 39.89 + 38.50 in the intervention group, 0.71 + 24.76 in the control group, *p* = 0003.	Not reported
Gowda et al. [19], 2015	Randomized, open label controlled study	Patients 18–60 years with DF or DHF I, II with platelet count 30-100 × 10^9^ (14 intervention group, 16 control group)	CP leaf extract tablets 1100 mg three times a day for five days	Mean platelet count on day 5: intervention group 104.71 ± 30.57, control group 66.63 ± 22.49. Mean difference in platelet count from day 1 to day 5: intervention group 39.92 ± 38.51, control group 00.69 ± 24.75, p = 0003.	Not reported
Gadhwal et al. [20], 2016	Randomized controlled study	Patients > 16 years with DF and platelet count < 150 × 10^9^ (200 in each group)	CP leaf extract capsule 500 mg once daily for five days	Mean platelet count was higher in the intervention group from 3rd to 5th day. Average hospitalization period: intervention group 3.65 ± 0.97 days, control group 5.42 ± 0.98, *p* < 0.01. Platelet transfusion requirements: intervention group 55/200, control group 93/200.	No side effects reported with CP
Kasture et al. [21], 2016	Randomized double blind placebo controlled study	Patients 18–60 years with DF or DHF I, II with platelet count 30-100 × 10^9^ (150 each group)	CP leaf extract tablets 1100 mg three times a day for five days	Median platelet count on day three: intervention group 88.89, control group 55.63; on day four 102.57 and 64.58; on day five 155.88 and 70.52, respectively. White cell count also increased in the intervention group. No difference in haematocrit.	Nausea, vomiting
Adarsh et al. [22], 2017	Randomized double blind placebo controlled study	Adult patients with DF (50 each)	CP leaf extract capsules 500 mg three times a day for five days	Average platelet count was higher in the intervention group on day 3, 4 and 5 (p < 0.01). Platelet transfusion requirements: intervention group 28%, control group 46% (p < 0.01). White cell count increased in the intervention group. Haematocrit did not show significant difference. Duration of hospitalization: intervention group 3.45 ± 0.98, control group 6.42 ± 0.98 (p < 0.01).	Nausea, vomiting
Srikanth et al. [23], 2019	Randomized open label controlled study	Paediatric patients (1–12 years) with DF or DHF grades I or II with platelet count 30-100 × 10^9^ (145 intervention, 140 control)	CP leaf extract syrup 275 mg three times a day (1–5 years age) or 550 mg three times a day (> 5 years)	Increase in platelet count on day 03, 04 and 05 were statistically significant (*p* < 0.05). Increase in red cell count was statistically significant on day 05 and white cell count statistically significant on day 3, 4 and 5.	Nausea in two patients

*CP Carica papaya*, *DF* dengue fever, *DHF* dengue haemorrhagic fever

One of the earliest randomized trials comes from Pakistan, where the effect of CP leaf extract on platelet counts was studied in 39 patients with DF or DHF [15]. The diagnosis of DF was based on WHO definitions, and only patients with platelet count< 50 × 10^9^/L were included, but inclusion and exclusion criteria were not well defined, and measures to minimize bias were not described. Increments in mean platelet counts daily and at the end of intervention were similar in those treated with CP and controls.

A randomized open-label controlled study from Indonesia tested the effect of CP leaf extract capsule (CPC) in patients with dengue fever [16]. Patients in the intervention group were treated with CPC 550 mg two capsules three times a day. Outcome measures were rise in platelet count, change in haematocrit, and period of hospitalization. The method of randomization and patient selection, and the day of inclusion, were not clearly described in this study. This study shows many methodological flaws that can increase the risk of bias, and in particular, the diagnosis of dengue was based on clinical grounds (fever, thrombocytopaenia and haemoconcentration) without laboratory confirmation. Platelet counts rose more rapidly in the intervention group (day 3) compared to the control group (day 5), and platelet counts on day 3 were significantly higher in the intervention group than counts on day 5 in the control group. Haematocrit values were similar in both groups. The period of hospitalization was shorter in the intervention group compared to the control group (3.48 ± 0.6 days *vs*. 5.38 ± 0.67 days, *p* < 0.05).

An open-label randomized controlled study from Malaysia assessed the mean platelet count difference in dengue patients after treating with CP leaf extract juice for three days [17]. Although 145 patients were included in each group, only 111(CP) and 119(controls) patients completed the study, increasing the risk of attrition bias. The study methodology was otherwise sound and well described, indicating measures to minimize bias in selection and randomization. Baseline mean platelet counts were similar in the two groups. Mean platelet counts from 8 h up to 40 h were not significantly different between the two groups. However at 40 h those who received CP had significantly higher platelet counts.

A randomized controlled study from India investigated the effect of CP leaf extract tablets on mean platelet counts in patients with DF and mild DHF [18]. In this study, the method of randomization, study participant selection, and day of febrile illness on which the intervention was started were not described. Mean platelet counts five days after commencing treatment rose in the intervention group compared to the control group (mean difference in platelets 39.89 + 38.50 × 10^9^/L in the intervention group *vs.* 0.71 + 24.76 × 10^9^/L in the control group, *p* = 0.003).

Another open-label randomized controlled study from India assessed the effect of CP leaf extract tablets on platelet counts in patients with dengue [19]. The method of randomization and exact selection methodology was not described in the study. Mean platelet counts on day 5 after treatment rose in the intervention group compared to controls.

In a larger open-label randomized controlled study from India, 400 patients were randomized to assess the effect of a lower dose of CPC in DF patients [20]. Simple randomization (odd-even method) was used. Apart from mean platelet counts, the other outcome measures were period of hospitalization and platelet transfusion. The indications for platelet transfusion were platelet count < 20 × 10^9^/L, bleeding tendency, and platelet count < 30 × 10^9^/L with decreasing trend. From the third day onwards, mean platelet counts were higher in the intervention group (day 3: CP 82.96 ± 16.72 × 10^9^/L, controls 66.45 ± 17.36 × 10^9^/L, *p* < 0.01; day 4: CP 122.43 ± 19.36 × 10^9^/L, controls 88.75 ± 21.65 × 10^9^/L, p < 0.01; day 4: CP 122.43 ± 19.36 × 10^9^/L, controls 88.75 ± 21.65 × 10^9^/L, p < 0.01; day 5: CP 112.47 ± 17.49 × 10^9^/L controls 102.59 ± 19.35 × 10^9^/L, p < 0.01). The mean period of hospitalization, and platelet transfusion requirements, were lower in those treated with CP.

The first double-blind placebo controlled study on CP in DF was a multicenter trial from India [21]. The methods of randomization and selection were not described. Outcome measures were changes in platelet counts, white cell counts, red cell counts and haematocrit. Median values for platelet counts in the CP group on third, fourth and fifth days were 88.897 × 10^9^/l, 102.579 × 10^9^/L and 155.886 × 10^9^/L, respectively. In the control group, median platelet counts were 55.633 × 10^9^/L, 64.582 × 10^9^/L and 70.528 × 10^9^/L respectively. White cell counts were also higher in the CP group. Haematocrit and red cell counts were similar in the two groups.

Another double-blind randomized controlled study from India evaluated the effects of CP leaf extract in DF [22]. This study also does not provide adequate information on methods to minimize bias. Outcome measures included platelet counts and other haematological parameters, duration of hospital stay, and platelet transfusion requirements. Platelet counts were higher in the CP group from the third to fifth day (*p* < 0.01). Similarly, white cell counts were higher in the CP group (*p* < 0.01). Haematocrit values were similar in the two groups. Platelet transfusions were according to WHO guidelines, and platelets were transfused in 28% of patients in the intervention group and 46% in the control group (p < 0.01). The period of hospitalization was longer in the control group (p < 0.01).

In a recent open-label randomized controlled study, the effect of CP leaf extract syrup was studied in 285 dengue patients aged less than 2 years [23]. Information on measures to minimize bias was limited. Although nine patients were excluded from efficacy analysis, reasons for this were given for only two. The primary outcome measure was mean increase in platelet count, while changes in red and white cells were taken as secondary outcome measures. Mean platelet counts were higher in the intervention group compared to the control group (*p* < 0.05).

**Fig. 3:**
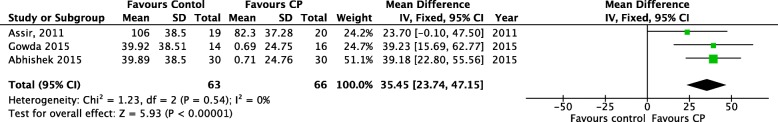
Forest plot of three studies comparing mean difference of platelet count between fifth and first day between two groups

Meta-analysis for the outcome measure of platelet recovery was possible for only three studies [15, 18, 19] (Fig. 3), because of heterogeneous methods used to determine platelet recovery, and inadequate data provided in other studies. Mean rise in platelets between the first and fifth days was significantly higher in the intervention group (mean difference 35.45, 95% confidence interval 23.74 to 47.15, 129 participants, low quality evidence).

**Fig. 4:**
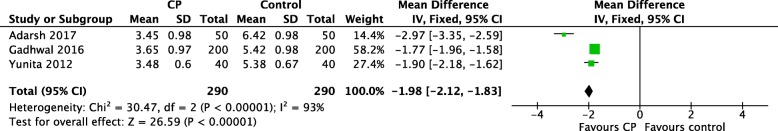
Forest plot of three studies comparing period of hospitalization between two groups

Three studies, involving 580 patients, also provided sufficient data on the duration of hospital stay [16, 20, 22] (Fig. 4). Pooled analysis showed that hospital stay was reduced in patients receiving papaya extract compared to either placebo or no treatment (Mean difference − 1.98 days, 95% confidence interval − 1.83 to − 2.12, 290 participants, low quality evidence).

Five studies reported on adverse events; three studies reported nausea and vomiting, [21–23] and two reported that there were no adverse events seen [15, 20]. The other studies did not mention details of adverse events.

## Discussion

Overall, considerable heterogeneity exists in controlled trials of CP extract in dengue published so far. There are marked variations in the enrollment criteria, categorization of patients, and measurements used to determine effects on platelet counts. Studies are mostly of low to moderate quality, with high risk of bias. The doses of CP used are variable, and no attempts have been made to determine effective dose. There are ethical concerns regarding the use of CP leaves or leaf extract without formal phase I testing, as while the fruits are edible, leaves are not routinely ingested. Nonetheless, extracts of CP leaves have not been shown to contain overtly toxic substances [9–11]., and comparative studies do not report significant adverse events with CP extract.

Given these limitations, there is some evidence of beneficial effects of CP in patients with dengue in reducing overall hospital stay. Platelet counts appear to improve more rapidly following treatment with CP, and in several studies the platelet counts at day 3–5 seem to be higher in those who received CP compared to those who did not. The shorter duration of hospital stay may be linked to improvement in platelet counts as well as clinical improvement, although the details of other parameters which might have prompted early discharge in these patients have not been studied in depth in any of the studies.

No studies examined the impact on mortality with CP. The effect of CP on the development or improvement of plasma leakage and haemorrhage has also not been studied, and the studies are either underpowered, or not designed with these important complications as outcome measures. In clinical practice, platelet levels correlate only loosely with the more dreaded complication of plasma leakage and shock syndrome, and the real test of efficacy of any therapeutic modality would be their effect on these life-threatening complications.

Nonetheless, there appears to be sufficient evidence from basic sciences research, and from current comparative clinical trials to justify conducting a large scale randomized double-blind placebo controlled trial to evaluate the potential benefits of CP extract. Such a trial should be designed, and powered, to determine the effects on CP on the following: preventing plasma leakage (for which platelet count changes may be a surrogate marker), prevention (or amelioration) of shock and multi-organ dysfunction, requirements for blood and blood product transfusion, the need for ICU admission, and the duration of ICU and hospital stay.

## Conclusions

Current clinical evidence on the beneficial effects of *Carica papaya* extract in the treatment of dengue infection is limited, and is based on a few trials of low to moderate quality. Improvements in platelet counts and reduction in hospital stay have been demonstrated, however significant methodological flaws in many of the studies makes it difficult to make clear recommendations. The routine use of CP extract for treating dengue cannot be recommended based on current available evidence.

## Data Availability

The datasets used and/or analysed during the current study are available from the corresponding author on reasonable request.
